# On the Condition of the Stomach and Intestines in Scarlatina

**Published:** 1865-01

**Authors:** Samuel Fenwick

**Affiliations:** Late Lecturer on Pathological Anatomy at the Newcastle-on-Tyne College of Medicine, in connection with the University of Durham


### ON THE CONDITION OF THE STOMACH AND INTESTINES IN SCARLATINA.

By Dr. SAMUEL FENWICK, late Lecturer on Pathological Anatomy at the Newcastle-on-Tyne College of Medicine, in connection with the University of Durham.

The object of this paper is to prove the following propositions:

1st. That the mucous membrane of the œsophagus, stomach, and intestines is inflamed in scarlatina.

2d. That desquamation of the epithelium of these parts takes place.

3d. That notwithstanding the anatomical changes in the mucous membrane of the stomach, the formation of pepsine is not prevented.

4th. That the condition of the skin is similar to the condition of the mucous membrane in scarlatina.

In support of the first proposition, the microscopic examinations of the mucous membranes of the œsophagus, stomach, and intestines were detailed in ten cases of death from scarlatina during the first week of illness, and in six cases who died in the second and third week of the fever. The first effects of the scarlatina poison upon the mucous membrane of the stomach were shown to be the congestion of the bloodvessels, and the stripping of the epithelium from the tubes and the surface of the organ, and also the softening of the tissues. The tubes are greatly distended by granular and fatty matters, or by small cells intermixed with granules, and in some cases they are lined by a newly-formed membrane. Sometimes no normal cells can be distinguished; in other cases they are present, but are scattered irregularly. After the second or third week the tubes are found less distended than at an earlier period, and whilst their closed ends are still loaded with granular matters, which greatly obscure the gastric cells, these become more evident towards the surface of the mucous membrane. The cells at this period are sometimes very large, sometimes loaded with fat or coated with granules, and seem to have but little adhesion to their basement membrane, as they readily separate from the tubes, but adhere closely to each other. The effects of the inflammation upon the intestines seem, in slighter cases, to consist in the effusion of granular and fatty matters into the mucous membrane; but in more severe cases the tubes of Lieberkühn are obstructed by epithelial cells, whilst extravasations of blood take place in the villi, and these with the rest of the mucous membrane, are loaded with small cells and granules. In one case the mucous membranes was entirely stripped of villi, excepting a few fragments which still remained, and the enlarged and prominent opening of the follicles of Lieberkühn gave its surface the appearance of a sieve. In some instances in which the pancreas has been examined, evidences of disease presented themselves.

The second proposition was stated to be more difficult of proof, inasmuch as vomiting usually occurs only in the first stage, and the author had no opportunity of examining the vomitted matters at this period of the disease. In one case, in which vomiting took place in the third week, fibrinous casts of the stomach tubes were discovered, and inflammation of the mucous membrane was proved to have existed by *post mortem* examination. The chief reason upon which the opinion that desquamation of the epithelium occurs was founded, was from the microscopic examination of the contents of the stomachs of those who had died of this disease. The contents in recent cases consisted of pieces of fine membrane, of cells, and of granules and shreds of membrane. The membranes were of the shape and size of the tubes of the stomach, and were covered with granules and fat. The cells varied from 1-1200th to 1-2200th of an inch, and were usually fringed with fine pieces of membrane. In cases of longer duration the membranes were covered with cells, and were also of the size and shape of the stomach tubes. In order to ascertain if these appearances were trustworthy as evidences of inflammation, the contents of the stomachs of forty-five subjects were examined at the Middlesex

Hospital, the condition of the mucous membrane being at the same time noted. In only one were there any fibrinous casts, and it was in a case of acute gastritis. In eighteen there were only separate cells, chiefly of the columnar form, and in none of these was there any inflammatory action. In eight cases casts of the upper parts of the tubes were plentiful, composed only of healthy conical cells, and in all the mucous membrane was in a natural condition. In eighteen there were either plugs formed of cells and granules from the secreting parts of the tubes, or the casts of conical cells were overlaid with granular matters, and in all of these the stomach was more or less inflamed. Two cases of gastritis, unconnected with scarlatina, were also quoted as examples of the forms in which casts of the stomach tubes appeared in vomited matters during life, and the author stated he had detected casts of the stomach tubes in matters vomited by persons affected with gastritis connected with diseased kidneys, with inflammatory dyspepsia, and other forms of inflammation of the gastric mucous membrane. It was urged that if casts of the gastric tubes can be discovered during life in cases of gastritis, and if in scarlatina this condition exists, and casts have been found in the stomach after death, there is every probability that desquamation of the epithelium takes place in this organ, as it does in the skin and the kidneys.

In support of the third proposition, the results of the following experiments were given in the three cases of scarlatina:—Ten grains of hard boiled white of egg were digested at a temperature of 90° for twelve hours in an infusion of the mucous membrane, to which three per cent. of hydrochloric acid had been previously added. The average loss of albumen was three grains and two-thirds. Similar experiments performed with the stomachs of eleven males who died of various diseases at the same hospital gave an average loss of four grains; so that there had been scarcely any diminution of pepsine produced by the fever. As a contrast to this were the results of similar experiments upon four cases who died of typhus fever. In two of these the albumen had gained three grains of weight by imbibition, and was not at all softened; whilst in the other two it was softened, and one had lost only half a grain, the other one grain and a-half in weight. But as the activity of the digestion must depend not only upon the relative amount of pespine, but also upon the bulk of the mucous membrane, this was also attempted to be estimated. The average weight of the mucous membrane of the stomachs of ten males dying of various diseases at the Middlesex Hospital was eighteen drachms, the weight of two recent cases of scarlatina was eighteen and sixteen drachms (the latter being in a boy), whilst it only amounted to fifteen drachms in one who died in the third week of illness. In four cases of typhoid fever the average weight of the mucous membrane only reached eleven drachms.

Under the fourth proposition, it was stated that the skin had only been examined microscopically in three cases. In the first, in which the patient died after a few days' illness, the only morbid appearance in the cutis was an occasional minute extravasation of blood in the neighborhood of the sudoriferous ducts. The rete mucosum was greatly thickened, and numerous round cells with large nuclei were everywhere visible, intermixed with the natural cells. The basement membranes of the sweat-glands were thickened, and the epithelium lining them was so much increased that in most cases it obstructed their channels. In some of the sweat-glands the coils of which they were composed were loaded with coagulated blood, and were greatly and irregularly distended. In the other recent case the appearances were similar, excepting that the external layers of the cuticle were stained with blood in minute patches, and the sweat-ducts were also reddened; but there were no extravasations of blood either in the glands or cutis. In some of the ducts the epithelium was detached from the basement membranes. In the case of a man who died during the third week, the sudoriferous tubes were still choked up, but in the glands the epithelium seemed in many places to be torn away, leaving the basement membranes bare, or only covered by ragged particles. The cutis was in a natural condition.

The author stated that although he had, in accordance with the usual custom, described the appearance of the skin and mucous membranes as the results of inflammation, yet that certain considerations suggested the idea that the term when so used was perhaps misapplied. In scarlatina, we find that in each part the morbid condition is mostly confined, in the first instance, to the basement membranes, and consists in the formation of layers of new cells, which, in the skin, are transformed into cuticle of natural appearance, and in the stomach contain pepsine. If future researches should prove that a similar condition occurs in the kidneys and other parts, it will be necessary to look upon the structural changes produced as resulting from increased physiological, rather than pathological action; and that the primary effect of the scarlatina poison is suddenly and violently to stimulate the natural cell-growth of the various secreting organs.—*Lancet*, *July* 23, 1864, *p*. 93.

### ON THE TREATMENT OF FEVER.

By DR. GULL, Physician to Guy's Hospital..

[The following short extract from some editorial remarks made by Dr. Gull, is contained in the "Reports of Hospital Practice" in the Medical Times and Gazette. When the pulse in the early stage of typhus or typhoid is very frequent, the patient requires to be well supported, in order to carry him through the later stages of the fever.]

Dr. Gull proceeded to remark: I do not believe in giving enormous doses of brandy in fever. My experience is against that system. I am sure of this, that if you have a severe case of typhus, and the patient is likely to die, no amount of brandy will save him.

Another part of the treatment that requires your attention is this: when I am called in to see a case of fever, I am not unfrequently told that the patient has been given something every half hour. In one case they said every five minutes. The plan of continually feeding Dr. Gull very strongly condemned; further saying, it is a very doubtful question in my mind whether much, or if any, of the nourishment given in severe cases is assimilated. But there is another way to support the patient; keep him cool, sponge his skin over three or four times a-day, give him plenty of liquids, to support the renal respiration, and plenty of fresh air to support the pulmonary respiration.—*Med. Times and Gazette, Aug.* 20, 1864, *p.* 196.

